# Ribosome biogenesis: Achilles heel of cancer?

**DOI:** 10.18632/genesandcancer.14

**Published:** 2014-05

**Authors:** Marjolein van Sluis, Brian McStay

**Affiliations:** Centre for Chromosome Biology, School of Natural Sciences, National University of Ireland Galway.

It is long been known that cancer and non-cancer cells can be distinguished on the basis of their nucleolar morphologies. As early as the 19^th^ century, it was reported that cancer cells have larger and more irregularly shaped nucleoli. Since then, pathologists have used nucleolar morphology to predict the clinical outcome [1]. Nucleolar morphology is altered due to the up-regulation of ribosomal gene transcription. Within nucleoli, ribosomal genes (rDNA) are transcribed by RNA polymerase I (pol I). The pre-ribosomal RNA (pre-rRNA) transcripts are subsequently modified and processed into the mature 18S, 5.8S and 28S rRNAs. 5S rRNA is transcribed by RNA polymerase III in the nucleoplasm. Together with the ribosomal proteins, the 5S rRNA is imported into the nucleolus where 40S and 60S ribosomal subunits are assembled prior to export to the cytoplasm [1, 2].

Oncogenes such as c-Myc can both directly and indirectly upregulate rDNA transcription, while tumour suppressors like p53 and Rb suppress ribosome biogenesis. Mutations in these genes not only result in deregulated cell cycle control, but also upregulated ribosome biogenesis. In addition to ribosome biogenesis, the nucleolus is a key cellular stress sensor and plays a central role in p53 activation [1, 2].

The increased translational capacity of cancer cells enables them to maintain higher proliferation rates. As stated by Ruggero, “compared with normal cells, cancer cells may be *addicted* to increases in ribosome biogenesis and number” [1]. This provides new therapeutic opportunities. As it turns out many chemotherapeutic drugs used in cancer treatment already inhibit ribosome biogenesis. In one recent survey it was shown that 20 out of 36 drugs in clinical use inhibit ribosome biogenesis [3]. Most of these drugs were originally designed to target highly proliferating cells by damaging DNA, interfering with DNA synthesis or with mitosis. These targeting modalities of these drugs also lead to toxicity in normal highly proliferating tissues. An example is ActinomycinD (AMD), a DNA intercalator which has a preference for GC-rich DNA sequences. As rDNA has above average GC-richness and because of its open chromatin conformation, low concentrations of AMD preferentially inhibit RNA polymerase I transcription and upon prolonged exposure causes genome wide DNA damage. Alkylating drugs like cisplatin and oxaliplatin or topoisomerases poisons like camptothecin inhibit pol I transcription. The degree to which inhibition of ribosome biogenesis contributes to the efficacy of these drugs is difficult to establish [3]. This raises an important question. Can targeting ribosome biogenesis without DNA damage offer any therapeutic potential? Two recently described drugs CX-5461 and BMH-21 are now providing evidence that inhibition of ribosome biogenesis by targeting transcription of rDNA by pol I has promising therapeutic potential. CX-5461 was designed to specifically inhibit pol I transcription by disrupting pre-initiation complex formation at the rDNA promoter. CX-5461 has been shown to activate p53 via nucleolar stress. It induces autophagy as well as senescence in a multiple types of cancer cells in a p53-dependent manner. Especially in leukaemia and lymphoma cells, treatment with CX-5461 induces p53-dependent apoptosis, while normal cells tolerate it [4, 5].

Whether the drug also induces DNA damage was not fully addressed, but it was demonstrated that it could induce cell death in cells lacking ATM - a key mediator of DNA double strand break responses. However, more recently Laiho and colleagues have shown that at high concentrations, CX-5461 does induce a γH2AX response, raising concerns about DNA damage [6].

BMH-21 was identified in a screen performed by Laiho and colleagues aimed at identifying novel p53 activators. Like AMD, BMH-21 is a DNA intercalator with preference for GC rich sequences [7]. Continuing the parallel with AMD, BMH-21 is a potent and specific inhibitor rDNA transcription and induces nucleolar reorganisation often referred to as nucleolar capping. Interestingly, transcription inhibition was followed by the degradation of the main pol I subunit, RPA194, by the proteasome [6]. In contrast with AMD, initial indications were that BMH-21 did not appear to induce DNA damage as evidenced by the lack of a γH2AX response [7]. Inhibition of transcription by BMH-21 causes nucleolar stress, resulting in decreased proliferation and cell death. P53 is activated in BMH-21 treated cells but is not required for its anti-proliferative effects. Intriguingly, it appears that cancer cells with high demands for ribosome biogenesis are selectively targeted [6].

The current publication in Oncotarget now rules out any role for DNA damage signalling and repair pathways in the BMH-21 response. Moreover, BMH-21 derivatives that can induce DNA damage display lower efficiency in inducing nucleolar stress and inhibiting proliferation [8]. The central importance of this study is that it finally uncouples DNA damage and nucleolar stress and reveals an Achilles heel in cancer cells, their addiction to ribosome biogenesis.
